# Integrating Polygenic Scores with Clinical, Lifestyle, and Social Risk Factors to Improve Heart Failure Risk Prediction

**DOI:** 10.1142/9789819824755_0046

**Published:** 2026

**Authors:** Katie M. Cardone, Dokyoon Kim, Marylyn D. Ritchie

**Affiliations:** 1 Department of Genetics, University of Pennsylvania Perelman School of Medicine, Philadelphia, PA, USA; 2 Institute for Biomedical Informatics, University of Pennsylvania Perelman School of Medicine, Philadelphia, PA, USA; 3 Division of Informatics, Department of Biostatistics, Epidemiology, and Informatics, University of Pennsylvania Perelman School of Medicine, Philadelphia, PA, USA

**Keywords:** Heart Failure, Polygenic Score, Clinical Risk Score, Polyexposure Score, Integrated Risk Model

## Abstract

Heart failure (HF) is highly prevalent, high-burden disorder with its prevalence expected to increase. Early detection of HF can reduce morbidity and mortality; therefore, novel early detection methods are needed. Polygenic scores (PGS) can combine common variants across the genome and provide phenotype-specific risk scores. However, there are also many well-known, non-genomic risk factors of HF, in the clinical, lifestyle, and social determinant of health (SDOH) domains, and it is not clear how genetic and non-genetic risk factors collectively contribute to HF risk. To address this question, we assessed whether combining HF PGS with clinical, lifestyle, and SDOH risk factors improves risk prediction. Leveraging data from the *All of Us* Research Program (n = 22,275), clinical risk factors were aggregated into a clinical risk score (CRS) while lifestyle and SDOH risk factors were aggregated into a polyexposure score (PXS). Feature selection was conducted with LASSO regression and statistical significance thresholding from logistic regression models (p < 0.05). Features were included in the model if they were statistically significant and important in ≥ 95% of 1000 iterations. To assess model performance, logistic regressions with HF case/control status were conducted with each risk score individually, as well as integrated models. The integrated model (PGS + CRS + PXS) performed better than individual risk scores (AUROC = 0.763, AUPRC = 0.047, F1 score = 0.062, balanced accuracy = 0.683). To assess the validity of the CRS and PXS, an integrated model with the PGS along with clinical and exposure risk factors as independent features was also evaluated. Based on AUPRC and F1 score, this integrated risk model (PGS + CRS risk factors + PXS risk factors) performed better than the combining the PGS with the CRS and PXS (AUROC = 0.738, AUPRC = 0.047, F1 score = 0.066, balanced accuracy = 0.657). These findings demonstrate that integration of risk factors across multiple domains can improve HF prediction. Knowing that PGS combined with clinical, lifestyle, and SDOH risk factors is predictive of HF risk provides greater opportunity for the identification of individuals at risk of HF prior to disease onset with the goal of prevention or early intervention.

## 1. Introduction

### 1.1. Heart Failure Risk Factors

Heart failure (HF) is a significant burden on the population, as 6.7 million individuals in the United States over age twenty are affected. Due to an aging population and increased survival rates after diagnosis, prevalence is expected to rise by millions each decade^1^. In addition, HF mortality rates have been increasing since 2012^1^. Early detection of HF may decrease morbidity and mortality, through early implementation of guideline-directed medical therapy, the gold-standard treatment for HF^1–3^. Major cardiovascular disease (CVD) events can be prevented with early detection, so, novel methods must be developed to improve early detection of HF^4^.

To date, the leading risk factors for HF include older age, smoking, atrial fibrillation (AF), hypertension, ischemic heart disease, obesity, and diabetes mellitus^1^. Cardiovascular conditions are interrelated and share many clinical, environmental, lifestyle, and social determinant of health (SDOH) risk factors^4^. Hypercholesterolemia and hyperlipidemia can lead to atherosclerosis and development of CVD, so many CVD treatments aim to lower lipid levels^4^. These risk factors are assessed in clinical settings with lipid panels that measure high-density lipoprotein (HDL) cholesterol, low-density lipoprotein (LDL) cholesterol, and triglycerides^4^. Other medications aim to lower blood pressure, as high blood pressure is also a major risk factor for CVD^4^. Another clinical risk factor for CVD is diabetes (type I and type II), which is measured by elevated glucose and hemoglobin A1c (HbA1c) levels^1,4^.

In addition to clinical risk factors, there are many lifestyle-related risk factors for CVD, including smoking, lack of physical activity, and poor diet^4^. Modifying lifestyle has a major impact on CVD as it is known to slow or reverse progression^4^. Sedentary lifestyle and unhealthy diet can lead to obesity, which is measured by body mass index (BMI)^4^. Lifestyle and SDOH risk factors are often interconnected^4,5^. For example, low income and education level are associated with nutrition status^4,5^. In addition, neighborhood factors such as grocery store availability, park and sidewalk access, poorly kept up housing, vandalism, and graffiti can influence diet and physical activity^4^. Other SDOH factors that are associated with CVD are single-living status, neighborhood deprivation, social isolation, employment status, food insecurity, childhood adversity, living alone, social deprivation index, and census-based income^4–8^. There are also environmental risk factors for CVD, such as air pollution and ambient temperatures^1,5,9,10^.

Genomics also play a role in HF development. HF has a heritability of 34% when excluding cardiomyopathies, suggesting some genomic contribution^11^. Familial/Mendelian traits that are caused by variants in single genes such as cardiomyopathies and hypercholesterolemia significantly increase the risk of HF^12–14^. Besides these Mendelian genetic risk factors, many common and rare genetic variants have also been associated with non-Mendelian HF^15^.

### 1.2. Heart Failure Risk Prediction

Known risk factors can be leveraged to predict HF risk. For example, genetic testing of variants in single genes that contribute to familial cardiomyopathies and hypercholesterolemia is used for early detection^12–14^. However, not all HF is Mendelian; therefore polygenic scores (PGS) offer an opportunity to aggregate common variants across the genome and provide phenotype-specific risk scores^16^. PGS are the cumulative, mathematical aggregation of risk derived from the total contribution of variants across the genome^16^. PGS have been shown to be predictive at the population-level for complex traits such as CAD, AF, type II diabetes (T2D), breast cancer, schizophrenia, bipolar disorder, among other traits^17–26^. Additionally, recent studies have found that PGS is predictive of HF^15,27–29^. The clinical utility of PGS is an active area of investigation and discussion in the field, with important considerations such as interpretability, integration with existing clinical risk models, cost-effectiveness, equitable access across diverse populations, and the need for clinician education and infrastructure to support its implementation^26,30–36^

Other risk scores, such as clinical risk scores (CRS) and polyexposure scores (PXS), can be used to integrate non-genomic risk factors, such as clinical, lifestyle, environmental, and SDOH variables into predictive risk models^6,37,38^. CRS is the linear combination of clinical risk factors associated with a disease of interest^38^. Many CRS have been developed and validated to predict HF, utilizing known HF predictor variables including clinical conditions, lifestyle factors, medications, and other risk factors^38^. These models have been shown to be predictive of HF (highest area under the receiver operating curve (AUROC) = 0.87)^38^. Conversely, PXS linearly integrates lifestyle, environmental, and SDOH risk factors into a singular score^37^. A PXS in one recent study was shown to be predictive of T2D status (C-index = 0.762)^37^.

These risk scores have been shown to be individually predictive for various CVDs. Integration of PGS with non-genomic risk factors has previously improved predictive ability for CAD, T2D, and aortic stenosis^39,40^. Another study found that combining PGS, CRS, and PXS together improved T2D classification accuracy^37^. Leveraging genomic, electronic health record (EHR), and survey data, this study aims to identify whether integration risk factors across multiple domains can improve prediction of HF risk.

## 2. Methods

### 2.1. Data and Study Participants

The *All of Us* Research Program (AOU) is a longitudinal, cohort study based in the United States^41^. Participants provided informed consent for optional data collection, including blood sample collection for whole genome sequencing (WGS), access to electronic health records (EHR) and wearables, and completion of health-related surveys and physical measurements at the time of enrollment^41^. Data from version 8 (v8) was utilized in this study^41^.

### 2.2. Genotyping and Quality Control

AOU genome centers extracted DNA from blood samples, which were genotyped with an Illumina NovaSeq 6000 instrument and processed on the Illumina DRAGEN platform^41^. Following processing, samples were included with mean coverage ≥ 30x, genome coverage ≥ 90% at 20x, coverage of hereditary disease risk genes ≥ 95% at 20x, aligned Q30 bases ≥ 8 × 10^10^, cross-individual contamination < 3%, concordance with independently processed genotype array, and concordance between sex call and self-reported sex at birth^41^. During joint calling, additional sample-level quality control (QC) was conducted, including sample hard threshold flagging (number of single nucleotide polymorphisms (SNPs) < 2.4 million and > 5.0 million, number of variants not present in gnomAD 3.1 > 100K, and heterozygous to homozygous ratio (Het/Hom) > 3.3 (for SNPs and insertions and deletions (INDELs) separately)), and sample population outlier flagging (eight median absolute deviations (MAD) away from the median residual in deletion count, insertion count, SNP count, number of variants not in gnomAD 3.1, insertion to deletion ratio, transition to transversion ratio, or SNP or INDEL Het/Hom). Variant-level quality control was also conducted, excluding variants with no high-quality genotype (genotype quality (GQ) ≥ 20, depth of coverage (DP) ≥ 10, and allele balance (AB) ≥ 0.02 for heterozygotes), excess heterozygosity < 54.69, SNP quality (QUAL) score < 60, INDEL QUAL score < 69, > 100 alternate alleles, and variants that are likely artifacts (using the Variant Extract-Train-Score Filtering (VETS) algorithm)^41^. Finally, eight well-characterized control samples were included to validate the QC pipeline by calculating sensitivity and precision^41^.

### 2.3. Polygenic Score Calculation

PGS weights were generated using the largest HF genome-wide association study (GWAS) to date (n-individuals = 2,322,691, n-variants = 1,274,692)^15^. Weights were extracted from the PGS catalog (PGS005097) and applied to the AOU cohort using the PGSC-CALC pipeline, which adjusts for the confounding effects of genetically inferred ancestry by normalizing based on differences in population means and standard deviations^42–44^.

### 2.4. Phenotyping

Individuals were categorized as cases and controls for ICD-based phenotypes based on ICD-9 and ICD-10 mappings to PhecodeX, which represent meaningful phenotypes in statistical genetics^45^. The outcome, HF status, was defined by PhecodeX CV_424. T2D status, a predictor included in the CRS, was defined by PhecodeX EM_202.2 (Table 1). For both phenotypes, individuals had to have at least two instances of mapped ICD codes to be a case (rule of two), and zero instances to be a control^46^. Age at first diagnosis, represented by age at first HF ICD code, was computed for HF cases, while age at last data release (October 1^st^, 2023) was computed for HF controls. Reported sex at birth was encoded numerically, including only males and females.

Lab values were derived from the electronic health record (EHR) and were obtained from serum or plasma. Triglycerides, HDL cholesterol, LDL cholesterol, non-fasting glucose, HbA1c, systolic blood pressure (SBP), and diastolic blood pressure (DBP) were included in the CRS while BMI was included in the PXS (Table 1). Values were first filtered by measurement name. Many values were extremely abnormal so values ≥ 5 MAD away from the median were excluded, as well as values ≤ 0. Many individuals had multiple measurements, so the closest value before their calculated age (age at first diagnosis for cases, age at data release for controls) was retained. Individuals who did not have lab measurements before their coded age were excluded.

Lifestyle risk factors (smoking status, physical activity, and nutrition status), as well as SDOH risk factors (income level, highest achieved education level, neighborhood, and single-living status), were extracted to be included in the PXS (Table 1). This data was derived from surveys, which were taken once at recruitment. Income, education level, and physical activity were derived from one question. Conversely, twenty-four neighborhood questions were extracted and kept as separate features. Smoking status was derived from nine questions which were integrated into one variable, classifying individuals as nonsmokers (0), former smokers (1), or current smokers (2)^47^. Individuals had to respond as a non-smoker in all questions to be classified as a non-smoker. Individuals were classified as former smokers if they identified as a former smoker or the age they completely quit smoking was less than their coded age. Individuals who responded as a smoker in any question were classified as a current smoker. Answers for all questions were coded numerically, such that each variable reflected a positive association with HF. The questions, answers, and answer encodings for each variable are described in the supplementary material (Supplementary Table 1). Census-based income and social deprivation index (SDI) were also included in the PXS, which were calculated by AOU based on three-digit zip codes (Table 1). Physical activity may change after a HF diagnosis, so the closest value prior to computed age was utilized. Individuals who did not have a physical activity variable prior to their coded age were excluded.

All continuous variables in the CRS and PXS, including labs, measurements, census-based income, and SDI were normalized with inverse-normal transformation. Prior to integration, all variables were put on the same scale. Variables were downscaled to match the variable with the lowest number of categories in their risk score group. CRS variables were scaled to 0–1 to align with T2D status and PXS variables were scaled to 0–2 to align with smoking status. Individuals with missing data in any variable were removed.

### 2.5. Clinical Risk Score and Polyexposure Score Construction

Data was split into 70% train and 30% test splits. The training portion was used for feature selection and weight generation. It was split in half, so 35% of the dataset was used for logistic regressions and 35% was used for LASSO regression. Logistic regression was used to identify risk factors that were significantly associated with HF (p < 0.05), while LASSO regression was used to identify important risk factors in HF prediction. In these training splits, SMOTE was used to combat case/control imbalance and increase sample size^48^. In both regression models, the outcome was HF case/control status, and age and sex were used as covariates in the logistic regression. 1000 iterations of these splits were conducted (for both 70/30 training/testing split and 35/35 training split), using a different random seed each time. Sampling was conducted without replacement. Only risk factors that were significant and important ≥ 95% of iterations (95% confidence) were included in CRS and PXS generation. In the testing set, risk factors were combined into cohesive scores using a weighted sum, using effect sizes (betas) from logistic regressions as the weights. As a comparison, unweighted sum was assessed as well.

### 2.6. Model Evaluation

Model evaluation was conducted in the testing split using logistic regressions. The testing set was split in half, so 15% of the dataset was used for model training and 15% was used for model testing. Like before, 1000 iterations of these splits were conducted. SMOTE was again used in the training set^48^. Each risk score was tested individually as well as every possible grouping of scores. Models with individual risk factors were tested as a comparison to the CRS and PXS. This resulted in seventeen distinct models (Table 3). In each model, age and sex were utilized as covariates. Performance metrics used were AUROC, area under the precision-recall curve (AUPRC), F1 score, and balanced accuracy. Mean metrics across the 1000 iterations were computed.

## 3. Results

Eighteen risk factors were initially selected for inclusion in the study (Table 1). Eight clinical risk factors were selected for the CRS, while four lifestyle risk factors and six SDOH risk factors were selected the PXS (Table 1, Supplementary Table 1). Nutrition and single-living status were excluded due to high data missingness (Table 1). DBP, SBP, and seven neighborhood variables were excluded because they were not significant in ≥ 95% of logistic regression iterations (Table 1, Table 2, Supplementary Table 1). Glucose and HbA1c were excluded because they were not important in ≥ 95% of LASSO regression iterations (Table 1, Table 2). Additionally, triglyceride levels were excluded because they were neither significant nor important (Table 1, Table 2). After filtering, ten risk factors remained, including three clinical risk factors (HDL cholesterol, LDL cholesterol, and T2D) in the CRS, and three lifestyle risk factors (physical activity, smoking status and BMI) and four SDOH risk factors (income level, education level, neighborhood, and census-based income) in the PXS, including seventeen neighborhood variables (Table 1, Table 2, Supplementary Table 1).

Prevalence of HF was ~5.1% in AOU. Removing missing data decreased the sample size substantially. The sample size decreased from 406,513 (n-controls = 386,518, n-cases = 19,995) to 22,594 (n-controls = 22,275, n-cases = 319). Lab values and neighborhood variables had the lowest percentage of non-missingness, which contributed the most to the sample size decrease (Supplementary Table 2). The case count in particular dropped because individuals had to have lab values and physical activity survey answers before their first HF diagnosis to be included. However, SMOTE was used to increase the case count to match the number of controls in all splits except the final testing set.

**Figure 2:**
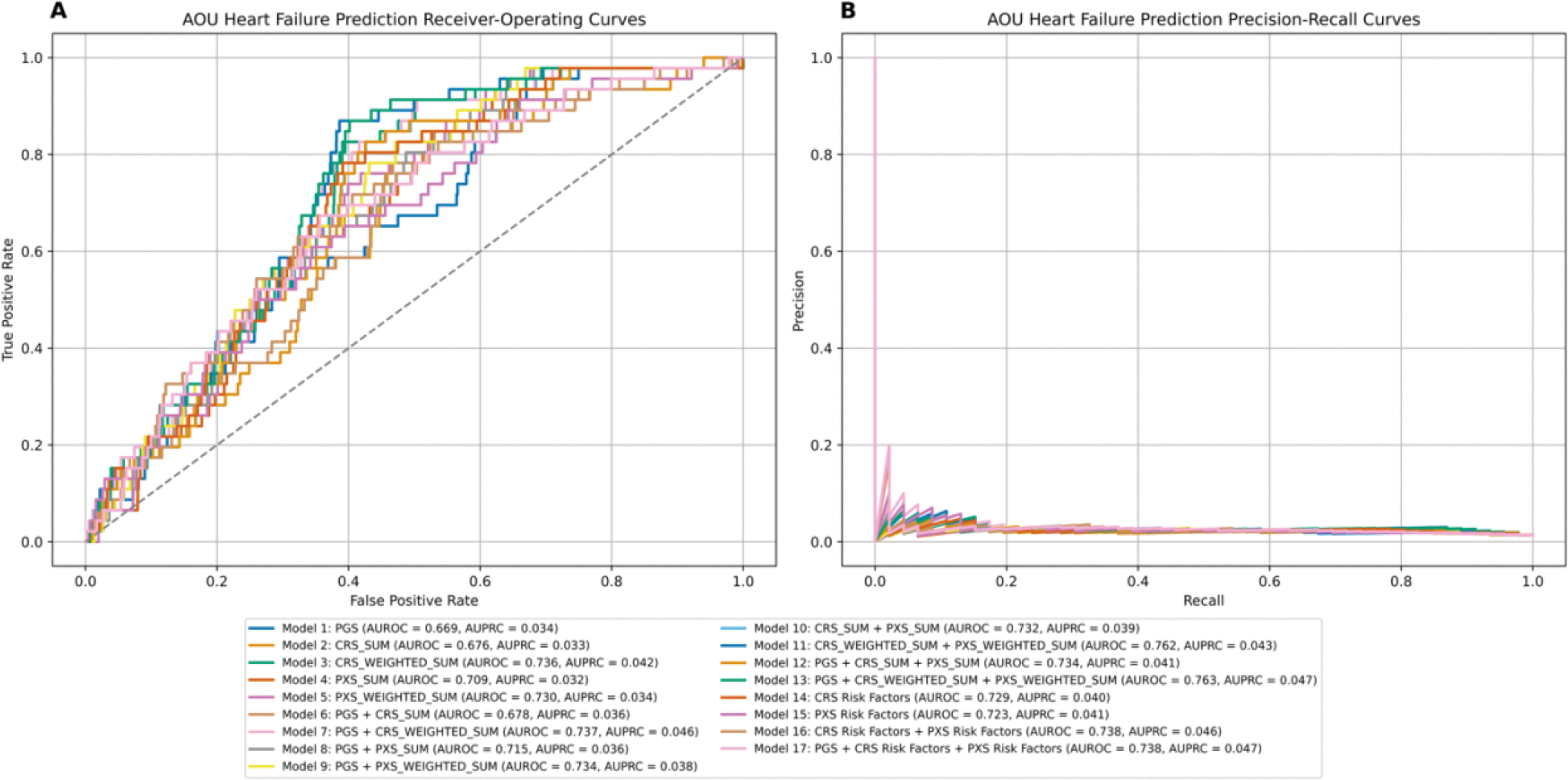
AOU Receiver-operator (ROC) curves (**Panel A**) and precision-recall (PRC) curves (**Panel B**).

Model 13 (PGS + weighted CRS + weighted PXS) performed best based on AUROC and balanced accuracy, while model 17 (PGS + CRS risk factors + PXS risk factors) performed best based on AUPRC and F1 score (Table 3, Table 4, Figure 2). Based on AUROC and balanced accuracy, the next best performing model was model 11 (weighted CRS + weighted PXS) (Table 3, Table 4, Figure 2). Based on F1 score and AUPRC, the next best performing models were model 13 (PGS + weighted CRS + weighted PXS) and model 16 (CRS risk factors + PXS risk factors) (Table 3, Table 4, Figure 2). After this, the performance ranking of the models varied by metric (Table 3, Table 4, Figure 2). However, based on AUROC, F1 score, and balanced accuracy, the worst performing models were model 4 (unweighted PXS), model 6 (PGS + unweighted CRS), model 2 (unweighted CRS), and model 1 (PGS) (Table 3, Table 4, Figure 2). Model 4 (unweighted PXS), model 2 (unweighted CRS), and model 1 (PGS) were among the worst performing models based on balanced accuracy, with the addition of model 5 (weighted PXS) (Table 3, Table 4, Figure 2).

## 4. Discussion

This study exhibited that integration of genetic, clinical, lifestyle, and SDOH risk factors improved predictive performance of heart failure risk in comparison to the separate risk scores alone. Based on all metrics, the integrated risk models containing risk factors across all domains (model 13, PGS + weighted CRS and PXS, and model 17, PGS + CRS and PXS risk factors) were the top performing models (Table 3, Table 4, Figure 2). Based on AUPRC and F1 score, model 17 (PGS + CRS and PXS risk factors) performed best but model 13 (PGS + weighted CRS and PXS) performed best based on AUROC and balanced accuracy (Table 3, Table 4, Figure 2). Thus, it is unclear which integration metric is best, but models containing risk factors across multiple domains were always the top performing model. This finding is consistent with previous literature^37,39,40^.

The PGS seemed to contribute the least to model performance, as model 1 (PGS) performed worse than model 3 (weighted CRS), model 14 (CRS risk factors), model 5 (weighted PXS), and model 15 (PXS risk factors) based on all metrics, which is consistent with previous literature (Table 3, Table 4, Figure 2)^37^. Additionally, model 11 (weighted CRS + weighted PXS) and model 16 (CRS risk factors + PXS risk factors) were among the top performing models. It is unclear whether clinical risk factors or lifestyle and SDOH risk factors contributed more, as performance ranking varied by metric, and quantitative differences were very small (Table 3, Table 4, Figure 2). In addition, models 3 (weighted CRS) and 5 (weighted PXS) performed better than models 2 (unweighted CRS) and 4 (unweighted PXS), demonstrating that integrating risk factor effect sizes enhances predictive performance.

Model 1 (PGS) yielded a lower AUROC compared to the study in which the weights were derived, possibly due to differences in the test dataset, sample size, or outcome phenotyping (Table 3, Table 4, Figure 2)^15^. Additionally, the PGSC-CALC pipeline normalized the PGS based on differences in population means and standard deviations, while the previous study did not normalize the PGS and instead scaled/centered the PGS within individual ancestry groups and utilized principal components as covariates in the regression models^15,42–44^. Models 2 (unweighted CRS) and 3 (weighted CRS) also had a lower AUROC than seen in previous literature (Table 3, Table 4, Figure 2)^38^. However, these studies included other clinical variables such as various clinical conditions, lab measurements, and medications, so it is possible that these variables in addition to different sample sizes, datasets, or combination methods, may have led to AUROC differences^38^. Model 5 (weighted PXS) has only been evaluated in one prior study, which did not use the same performance metrics, preventing direct pairwise comparison^37^.

Model training yielded variables that were considered most predictive. Based on logistic regressions, the ten risk factors with the lowest mean p-values were T2D, income, physical activity, BMI, census-based income, education, four neighborhood variables, and LDL cholesterol (Table 2). T2D, LDL, BMI, physical activity, and census-based income overlapped with the ten risk factors with the highest mean absolute coefficients based on LASSO regressions (Table 2). Other variables deemed more important by LASSO regressions were HDL cholesterol, DBP, SBP, triglycerides, and HbA1c (Table 2). SDOH risk factors appeared more significant in logistic regressions, while clinical risk factors appeared more important in LASSO regressions (Table 2). Thus, there is some variability based on the feature selection method.

This study had several limitations. Sample size was low due to removal of missing values, and performance may have improved if it was higher, particularly in the training sets. It is also possible that more predictors may have appeared significant and important in ≥ 95% of iterations, which could have changed performance. This study also did not include a validation dataset, and replication would have strengthened these findings. However, these findings are replicated by similar studies in the literature^37,38,40^. Several other factors may have improved performance. For example, performance may have improved with the inclusion more clinical, lifestyle, and SDOH risk factors associated with heart failure, as not all known HF/CVD risk factors were included in this study. CVDs have some overlapping genetic architecture, so correcting for this may have improved performance^49^. PGS performance was worse than risk factors in other domains, and it is possible that stratifying the sample to high-risk individuals classified by other CVD risk factors may improve its performance^26^. In addition, performance may have been enhanced with ancestry-stratified analyses, but the sample size was too low to further subset the cohort. As AOU continues to grow, we anticipate that sample sizes will increase and much of these risk factors will become populated in these datasets in the future. There are also some limitations regarding the phenotyping strategy. Rule of two phenotyping accounts for some error in ICD-based phenotyping, but it is possible that there may be some present errors as ICD codes are used for billing purposes and not diagnosis documentation^46^. In addition, many of the lifestyle and SDOH risk factors were based on self-reported survey data, which is susceptible to bias^50^. Last, prevalence of HF in the general population is estimated to be 1.9 – 2.8%, which is lower than prevalence in these datasets^1^. This, in addition to participation bias of individuals who opt in to biobank studies, may impact the generalizability of this study’s findings to the general population^51^.

Despite these limitations, this study demonstrated that integrating risk factors across multiple domains improves predictive performance of HF risk, highlighting the need to consider these risk factors in clinical settings. Integrating polygenic scores with clinical, lifestyle, and SDOH risk factors may be used to improve early detection of HF, and therefore its adverse consequences. Such multi-domain risk models may facilitate more precise risk stratification in clinical settings, enabling earlier interventions and improved management for individuals at risk for HF.

## Supplementary Material

suppl table

Supplementary Material

All supplemental material can be found at: https://ritchielab.org/publications/supplementary-data/psb-2026/hf-irm. All code can be found at: https://github.com/RitchieLab/HF_IRM.git

## Figures and Tables

**Figure 1:**
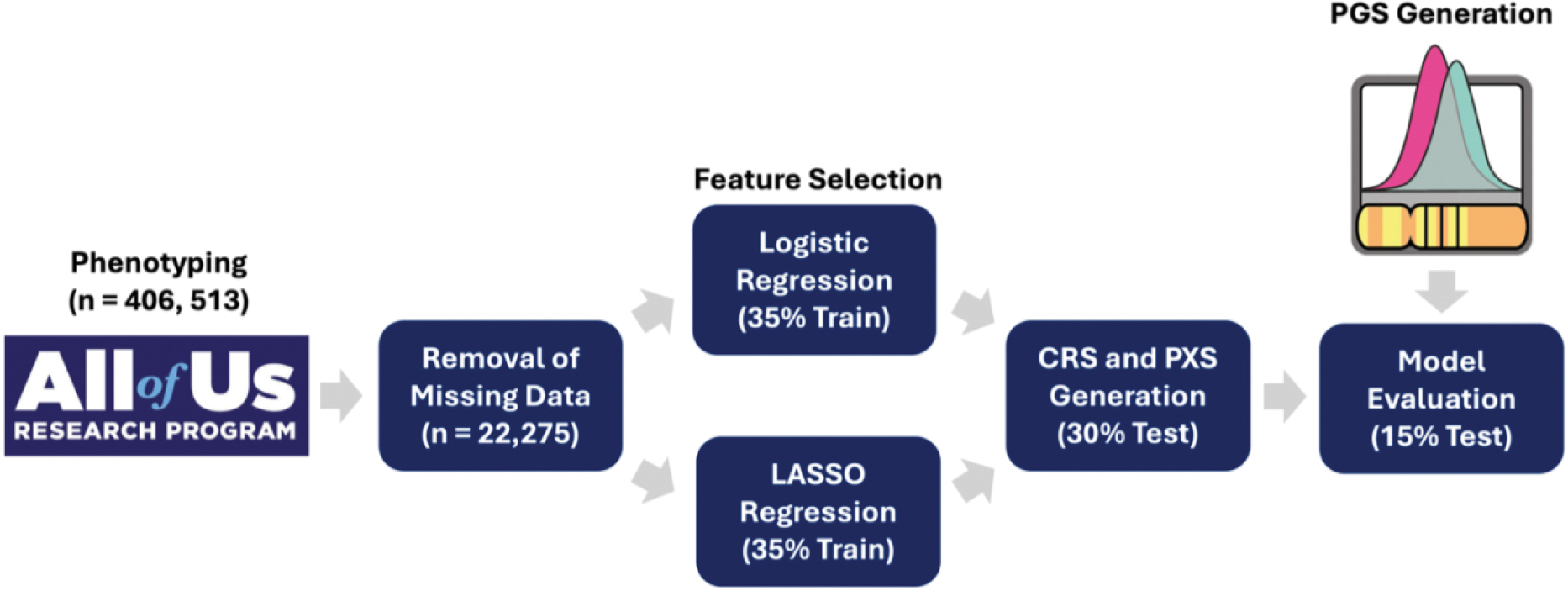
Overview of methodology

**Table 1:** Known risk factors for HF to be included in integrated risk scores.

Clinical Risk Score	Polyexposure Score	

Clinical Risk Factors	Lifestyle Risk Factors	Social Determinant of Health Risk Factors

Diastolic Blood Pressure^†^	BMI	Census-based income
Glucose^‡^	Nutrition status*	Education level
HbA1c^‡^	Physical activity	Income level
HDL cholesterol	Smoking status	Neighborhood^†^
LDL cholesterol		Single-level status*
Systolic Blood Pressure^†^		Deprivation index^†‡^
T2D status		
Triglycerides^†‡^		

* Risk factors excluded due to low data availability.

† Risk factors excluded because they were not significant in ≥ 95% of logistic regression iterations.

‡ Risk factors excluded because they were not important in ≥ 95% of LASSO regression iterations.

Some, but not all, neighborhood questions were excluded.

**Table 2:** Significance and importance of variables across 1000 iterations.

Category (Risk Score)	Risk Factor	Mean P-Value	Percent Significant	Mean Absolute Coefficient	Percent Important
Clinical (CRS)	Diastolic Blood Pressure	0.048	88.30%	1.322	96.30%
Clinical (CRS)	Glucose	0.007	97.80%	0.819	93.80%
Clinical (CRS)	HbA1c	0.001	99.70%	0.914	91.90%
Clinical (CRS)	HDL cholesterol	0.008	98.00%	2.014	97.70%
Clinical (CRS)	LDL cholesterol	0.000	99.90%	1.403	95.00%
Clinical (CRS)	Systolic Blood Pressure	0.112	73.50%	0.946	92.60%
Clinical (CRS)	T2D	9.50E-81	100.00%	0.980	100.00%
Clinical (CRS)	Triglycerides	0.120	71.40%	0.924	92.60%
Lifestyle (PXS)	BMI	3.82E-20	100.00%	2.511	100.00%
Lifestyle (PXS)	Everyday Physical Activity	4.40E-48	100.00%	0.847	100.00%
Lifestyle (PXS)	Smoking	0.004	99.10%	0.242	99.70%
SDOH (PXS)	Annual Income	1.16E-54	100.00%	0.397	99.40%
SDOH (PXS)	Census Median Income	3.40E-18	100.00%	0.914	96.90%
SDOH (PXS)	Highest Education	6.23E-08	100.00%	0.385	97.30%
SDOH (PXS)	Neighborhood- Abandoned Buildings	0.002	99.40%	0.293	97.10%
SDOH (PXS)	Neighborhood- Alcohol Use	0.023	94.30%	0.667	99.40%
SDOH (PXS)	Neighborhood- A lot of Crime	0.005	99.00%	0.365	98.00%
SDOH (PXS)	Neighborhood- Cleanliness	0.016	95.30%	0.288	97.90%
SDOH (PXS)	Neighborhood-Crime Rate Makes It Unsafe to Walk at Night	0.001	99.90%	0.343	99.00%
SDOH (PXS)	Neighborhood- Crime Rate Makes It Unsafe to Walk During the Day	1.95E-13	100.00%	0.544	99.00%
SDOH (PXS)	Neighborhood- Drug Use	0.003	99.40%	0.484	97.20%
SDOH (PXS)	Neighborhood- Facilities to Bike	2.47E-07	100.00%	0.324	99.40%
SDOH (PXS)	Neighborhood- Free/Low-Cost Recreation Facilities	0.001	99.20%	0.243	99.30%
SDOH (PXS)	Neighborhood- Get Along with Neighbors	0.113	73.00%	0.835	99.20%
SDOH (PXS)	Neighborhood- Graffiti	0.014	96.20%	0.301	97.90%
SDOH (PXS)	Neighborhood-Main Type of Housing	0.075	80.80%	0.256	98.30%
SDOH (PXS)	Neighborhood- Neighbors Can Be Trusted	0.001	99.90%	0.451	96.70%
SDOH (PXS)	Neighborhood- Neighbors Take Good Care of Their Homes	0.006	98.80%	0.256	97.80%
SDOH (PXS)	Neighborhood- Neighbors Watch Out for Each Other	0.046	88.00%	0.289	98.40%
SDOH (PXS)	Neighborhood- Noise	0.099	77.40%	0.255	97.70%
SDOH (PXS)	Neighborhood- People Share the Same Values	0.046	88.30%	0.293	97.90%
SDOH (PXS)	Neighborhood- Safe from Crime	0.002	99.40%	0.303	97.80%
SDOH (PXS)	Neighborhood- Shops, Stores, Markets or Other Places to Buy Things are Within Walking Distance	0.000	99.90%	0.309	99.40%
SDOH (PXS)	Neighborhood- Sidewalks on Most Streets	0.008	97.90%	0.159	98.70%
SDOH (PXS)	Neighborhood- Too Many People Hanging Around on the Streets Near Home	0.014	96.40%	0.345	96.60%
SDOH (PXS)	Neighborhood- Transit Stop Within Walking Distance	0.019	95.20%	0.152	98.70%
SDOH (PXS)	Neighborhood- Trouble with Neighbors	0.002	99.50%	0.347	97.70%
SDOH (PXS)	Neighborhood- Vandalism	0.006	98.50%	0.304	97.20%
SDOH (PXS)	Social Deprivation Index	0.090	77.60%	0.697	93.40%

Mean beta, mean p-value and percent significant columns were calculated from logistic regressions, while mean absolute coefficient and percent important columns were calculated from LASSO regressions.

**Table 3:** Overview of evaluation models.

Model Number	Model	Number of Features
1	PGS	3
2	CRS_SUM	3
3	CRS_WEIGHTED_SUM	3
4	PXS_SUM	3
5	PXS_WEIGHTED_SUM	3
6	PGS + CRS_SUM	4
7	PGS + CRS_WEIGHTED_SUM	4
8	PGS + PXS_SUM	4
9	PGS + PXS_WEIGHTED_SUM	4
10	CRS_SUM + PXS_SUM	4
11	CRS_WEIGHTED_SUM + PXS_WEIGHTED_SUM	4
12	PGS + CRS_SUM + PXS_SUM	5
13	PGS + CRS_WEIGHTED_SUM + PXS_WEIGHTED_SUM	5
14	CRS Risk Factors	7
15	PXS Risk Factors	12
16	CRS Risk Factors + PXS Risk Factors	17
17	PGS + CRS Risk Factors + PXS Risk Factors	18

Features included each risk score, as well as age and sex as covariates.

**Table 4:** Model evaluation metrics.

Model Number	Risk Factor	AUROC	AUPRC	F1 Score	Balanced Accuracy
1	PGS	0.669	0.034	0.044	0.607
2	CRS_SUM	0.676	0.033	0.042	0.598
3	CRS_WEIGHTED_SUM	0.736	0.042	0.055	0.655
4	PXS_SUM	0.709	0.032	0.052	0.646
5	PXS_WEIGHTED_SUM	0.730	0.034	0.056	0.660
6	PGS + CRS_SUM	0.678	0.036	0.043	0.603
7	PGS + CRS_WEIGHTED_SUM	0.737	0.046	0.057	0.662
8	PGS + PXS_SUM	0.715	0.036	0.054	0.652
9	PGS + PXS_WEIGHTED_SUM	0.734	0.038	0.057	0.665
10	CRS_SUM + PXS_SUM	0.732	0.039	0.055	0.652
11	CRS_WEIGHTED_SUM + PXS_WEIGHTED_SUM	0.762	0.043	0.061	0.678
12	PGS + CRS_SUM + PXS_SUM	0.734	0.041	0.057	0.658
13	PGS + CRS_WEIGHTED_SUM + PXS_WEIGHTED_SUM	0.763	0.047	0.062	0.682
14	CRS Risk Factors	0.729	0.040	0.055	0.655
15	PXS Risk Factors	0.723	0.041	0.060	0.647
16	CRS Risk Factors + PXS Risk Factors	0.738	0.046	0.065	0.658
17	PGS + CRS risk factors + PXS risk factors	0.738	0.047	0.065	0.657

## References

[1] Bozkurt B HF STATS 2024: Heart Failure Epidemiology and Outcomes Statistics An Updated 2024 Report from the Heart Failure Society of America. J. Card. Fail. 31, 66–116 (2025). 39322534 10.1016/j.cardfail.2024.07.001

[2] Wang H Importance of early diagnosis and treatment of heart failure across the spectrum of ejection fraction. Eur. Heart J. 44, ehad655.892 (2023).

[3] Kittleson MM 2024 Update to the 2020 ACC/AHA Clinical Performance and Quality Measures for Adults With Heart Failure: A Report of the American Heart Association/American College of Cardiology Joint Committee on Performance Measures. Circ. Cardiovasc. Qual. Outcomes 17, e000132 (2024). 39116212 10.1161/HCQ.0000000000000132

[4] 2025 Heart Disease and Stroke Statistics: A Report of US and Global Data From the American Heart Association | Circulation. https://www.ahajournals.org/doi/10.1161/CIR.0000000000001303. 10.1161/CIR.0000000000001303 PMC12256702 39866113

[5] Bazoukis G Impact of Social Determinants of Health on Cardiovascular Disease. J. Am. Heart Assoc. 14, e039031 (2025). 40035388 10.1161/JAHA.124.039031 PMC12132660

[6] Ana Palacio MD Social Determinants of Health Score: Does It Help Identify Those at Higher Cardiovascular Risk? 26, (2020). 10.37765/ajmc.2020.88504 33094943

[7] Jilani MH Social Determinants of Health and Cardiovascular Disease: Current State and Future Directions Towards Healthcare Equity. Curr. Atheroscler. Rep. 23, 55 (2021). 34308497 10.1007/s11883-021-00949-w

[8] Bevan GH, Nasir K, Rajagopalan S & Al-Kindi S Socioeconomic Deprivation and Premature Cardiovascular Mortality in the United States. Mayo Clin. Proc. 97, 1108–1113 (2022). 35300876 10.1016/j.mayocp.2022.01.018 PMC10411485

[9] Jia Y Effect of Air Pollution on Heart Failure: Systematic Review and Meta-Analysis. Environ. Health Perspect. 131, 76001 (2023). 37399145 10.1289/EHP11506 PMC10317211

[10] Feng J, Zhang Y & Zhang J Epidemiology and Burden of Heart Failure in Asia. JACC Asia 4, 249–264 (2024). 38660101 10.1016/j.jacasi.2024.01.013 PMC11035951

[11] Lindgren MP A Swedish Nationwide Adoption Study of the Heritability of Heart Failure. JAMA Cardiol. 3, 703–710 (2018). 29998296 10.1001/jamacardio.2018.1919 PMC6583873

[12] Miller DT ACMG SF v3.2 list for reporting of secondary findings in clinical exome and genome sequencing: A policy statement of the American College of Medical Genetics and Genomics (ACMG). Genet. Med. Off. J. Am. Coll. Med. Genet. 25, 100866 (2023). 10.1016/j.gim.2023.100866 PMC10524344 37347242

[13] Brownrigg JR Epidemiology of cardiomyopathies and incident heart failure in a population-based cohort study. Heart Br. Card. Soc. 108, 1383–1391 (2022). 10.1136/heartjnl-2021-320181 34969871

[14] Tada H Familial hypercholesterolemia is related to cardiovascular disease, heart failure and atrial fibrillation. Results from a population-based study. Eur. J. Clin. Invest. 54, e14119 (2024). 37916502 10.1111/eci.14119

[15] Lee DSM Common-variant and rare-variant genetic architecture of heart failure across the allele-frequency spectrum. Nat. Genet. 57, 829–838 (2025). 40195560 10.1038/s41588-025-02140-2 PMC12049093

[16] Choi SW, Mak TS-H & O’Reilly PF Tutorial: a guide to performing polygenic risk score analyses. Nat. Protoc. 15, 2759–2772 (2020). 32709988 10.1038/s41596-020-0353-1 PMC7612115

[17] Ratman D Polygenic risk scores improve CAD risk prediction in individuals at borderline and intermediate clinical risk. Npj Cardiovasc. Health 2, 13 (2025). 10.1038/s44325-025-00049-7 PMC12912298 41775875

[18] Patel AP A multi-ancestry polygenic risk score improves risk prediction for coronary artery disease. Nat. Med. 29, 1793–1803 (2023). 37414900 10.1038/s41591-023-02429-x PMC10353935

[19] Gibson JT & Rudd JHF Polygenic risk scores in atrial fibrillation: Associations and clinical utility in disease prediction. Heart Rhythm 21, 913–918 (2024). 38336192 10.1016/j.hrthm.2024.02.006

[20] Khera AV Genome-wide polygenic scores for common diseases identify individuals with risk equivalent to monogenic mutations. Nat. Genet. 50, 1219–1224 (2018). 30104762 10.1038/s41588-018-0183-z PMC6128408

[21] Ge T Development and validation of a trans-ancestry polygenic risk score for type 2 diabetes in diverse populations. Genome Med. 14, 70 (2022). 35765100 10.1186/s13073-022-01074-2 PMC9241245

[22] Roberts E, Howell S & Evans DG Polygenic risk scores and breast cancer risk prediction. Breast Edinb. Scotl. 67, 71–77 (2023). 10.1016/j.breast.2023.01.003 PMC9982311 36646003

[23] Mars N Polygenic and clinical risk scores and their impact on age at onset and prediction of cardiometabolic diseases and common cancers. Nat. Med. 26, 549–557 (2020). 32273609 10.1038/s41591-020-0800-0

[24] Duncan L Polygenic scores for psychiatric disorders in a diverse postmortem brain tissue cohort. Neuropsychopharmacology 48, 764–772 (2023). 36694041 10.1038/s41386-022-01524-w PMC10066241

[25] Liu H, Wang L, Yu H, Chen J & Sun P Polygenic Risk Scores for Bipolar Disorder: Progress and Perspectives. Neuropsychiatr. Dis. Treat. 19, 2617–2626 (2023). 38050614 10.2147/NDT.S433023 PMC10693760

[26] O’Sullivan JW Polygenic Risk Scores for Cardiovascular Disease: A Scientific Statement From the American Heart Association. Circulation 146, e93–e118 (2022). 35862132 10.1161/CIR.0000000000001077 PMC9847481

[27] Soh CH, Xiang R, Takeuchi F & Marwick TH Use of Polygenic Risk Score for Prediction of Heart Failure in Cancer Survivors. JACC CardioOncology 6, 714–727 (2024). 39479322 10.1016/j.jaccao.2024.04.010 PMC11520200

[28] Han Y A novel polygenic risk score improves prognostic prediction of heart failure with preserved ejection fraction in the Chinese Han population. Eur. J. Prev. Cardiol. 30, 1382–1390 (2023). 37343143 10.1093/eurjpc/zwad209

[29] Ahn H-J Polygenic risk-based prediction of heart failure in young patients with atrial fibrillation: an analysis from UK Biobank. EP Eur. 27, euaf104 (2025). 10.1093/europace/euaf104 PMC12212053 40388376

[30] Martin AR Clinical use of current polygenic risk scores may exacerbate health disparities. Nat. Genet. 51, 584–591 (2019). 30926966 10.1038/s41588-019-0379-x PMC6563838

[31] Abramowitz SA Evaluating Performance and Agreement of Coronary Heart Disease Polygenic Risk Scores. JAMA 333, 60–70 (2025). 39549270 10.1001/jama.2024.23784 PMC11569413

[32] Kumuthini J The clinical utility of polygenic risk scores in genomic medicine practices: a systematic review. Hum. Genet. 141, 1697–1704 (2022). 35488921 10.1007/s00439-022-02452-x PMC9055005

[33] Xiang R Recent advances in polygenic scores: translation, equitability, methods and FAIR tools. Genome Med. 16, 33 (2024). 38373998 10.1186/s13073-024-01304-9 PMC10875792

[34] Lewis CM & Vassos E Prospects for using risk scores in polygenic medicine. Genome Med. 9, 96 (2017). 29132412 10.1186/s13073-017-0489-y PMC5683372

[35] Torkamani A, Wineinger NE & Topol EJ The personal and clinical utility of polygenic risk scores. Nat. Rev. Genet. 19, 581–590 (2018). 29789686 10.1038/s41576-018-0018-x

[36] Lennon NJ Selection, optimization and validation of ten chronic disease polygenic risk scores for clinical implementation in diverse US populations. Nat. Med. 30, 480–487 (2024). 38374346 10.1038/s41591-024-02796-z PMC10878968

[37] He Y Comparisons of Polyexposure, Polygenic, and Clinical Risk Scores in Risk Prediction of Type 2 Diabetes. Diabetes Care 44, 935–943 (2021). 33563654 10.2337/dc20-2049 PMC7985424

[38] Sinha A Risk-Based Approach for the Prediction and Prevention of Heart Failure. Circ. Heart Fail. 14, e007761 (2021). 33535771 10.1161/CIRCHEARTFAILURE.120.007761 PMC7887083

[39] Small AM Novel Polygenic Risk Score and Established Clinical Risk Factors for Risk Estimation of Aortic Stenosis. JAMA Cardiol. 9, 357–366 (2024). 38416462 10.1001/jamacardio.2024.0011 PMC10902779

[40] van Dam S The necessity of incorporating non-genetic risk factors into polygenic risk score models. Sci. Rep. 13, 1351 (2023). 36807592 10.1038/s41598-023-27637-w PMC9941118

[41] Bick AG Genomic data in the All of Us Research Program. Nature 627, 340–346 (2024). 38374255 10.1038/s41586-023-06957-x PMC10937371

[42] Lambert SA Enhancing the Polygenic Score Catalog with tools for score calculation and ancestry normalization. Nat. Genet. 56, 1989–1994 (2024). 39327485 10.1038/s41588-024-01937-x PMC12041910

[43] Khera AV Whole-Genome Sequencing to Characterize Monogenic and Polygenic Contributions in Patients Hospitalized With Early-Onset Myocardial Infarction. Circulation 139, 1593–1602 (2019). 30586733 10.1161/CIRCULATIONAHA.118.035658 PMC6433484

[44] Khan A Genome-wide polygenic score to predict chronic kidney disease across ancestries. Nat. Med. 28, 1412–1420 (2022). 35710995 10.1038/s41591-022-01869-1 PMC9329233

[45] Shuey MM Next-generation phenotyping: introducing phecodeX for enhanced discovery research in medical phenomics. Bioinformatics 39, btad655 (2023). 37930895 10.1093/bioinformatics/btad655 PMC10627409

[46] Schrodi SJ The Impact of Diagnostic Code Misclassification on Optimizing the Experimental Design of Genetic Association Studies. J. Healthc. Eng. 2017, 7653071 (2017). 29181145 10.1155/2017/7653071 PMC5664372

[47] Tindle HA Lifetime Smoking History and Risk of Lung Cancer: Results From the Framingham Heart Study. J. Natl. Cancer Inst. 110, 1201–1207 (2018). 29788259 10.1093/jnci/djy041 PMC6235683

[48] Chawla NV, Bowyer KW, Hall LO & Kegelmeyer WP SMOTE: synthetic minority over-sampling technique. J Artif Int Res 16, 321–357 (2002).

[49] Qiao J Shared genetic architecture contributes to risk of major cardiovascular diseases. Nat. Commun. 16, 8368 (2025). 40993117 10.1038/s41467-025-62419-0 PMC12460838

[50] Choi BCK & Pak AWP. A Catalog of Biases in Questionnaires. Prev. Chronic. Dis. 2, A13 (2004). 15670466 PMC1323316

[51] Ridgeway JL Potential Bias in the Bank: What Distinguishes Refusers, Non-responders and Participants in a Clinic-based Biobank? Public Health Genomics 16, 10.1159/000349924 (2013). PMC3821039 23595106

