# Supplementary material for: Integrating Polygenic Scores with Clinical, Lifestyle, and Social Risk Factors to Improve Heart Failure Risk Prediction
Source: Pac Symp Biocomput. Author manuscript; Available in PMC 2026 Mar 2. (PMC12952681; doi:10.1142/9789819824755_0046)
Supplement: suppl table [file NIHMS2130997-supplement-suppl_table.pdf]

Supplementary Table 1: All of Us variables obtained from survey and observation data with their corresponding questions, answers, and encodings. *Smoking Encoding: 0 = non-smoker, 1 = former smoker, 2 = smoker

Category (Risk Score)	Variable Name	Question/Statement	Answer Encodings	Answer Encoding Range
**Lifestyle (PXS)**	Physical Activity- Everyday Physical Activity	To what extent are you able to carry out your everyday physical activities such as walking, climbing stairs, carrying groceries, or moving a chair [PROMIS]	0 = Not at all; 1 = A little; 2 = Moderately; 3 = Mostly; 4 = Completely, Missing = Skip	0-4
**Lifestyle (PXS)**	Smoking- ≥100 Cigarettes in Lifetime	Smoked at least 100 cigarettes in entire life	0 = No; 2 = Yes*	0-2
**Lifestyle (PXS)**	Smoking- Cigar Smoking Y/N	Cigar Smoking: Cigar Smoke Participant	0 = No; 2 = Yes*	0-2
**Lifestyle (PXS)**	Smoking- Cigarettes Per Day	How many cigarettes do you smoke per day now [PhenX]	0 = 0; 2 = > 0*	0-2
**Lifestyle (PXS)**	Smoking- Completely Quit Age	Attempt Quit Smoking: Completely Quit Age	1 = Quit age < coded age; 2 = Quit age > coded age*	1-2
**Lifestyle (PXS)**	Smoking- Current Cigar Frequency	Cigar Smoking: Current Cigar Frequency	0 = Not at all; 2 = Some days; 2 = Every day; Missing = Skip*	0-2
**Lifestyle (PXS)**	Smoking- Current Cigarette Frequency	Do you now smoke cigarettes every day, some days, or not at all [PhenX]	0 = Not at all; 2 = Some days; 2 = Every day; Missing = Skip*	0-2
**Lifestyle (PXS)**	Smoking- Number of Years	Smoking: Number Of Years	0 = 0; 2 = >0; Missing = Don't know; Missing = Skip; Missing = Prefer not to answer*	0-2
**Lifestyle (PXS)**	Smoking- Smoke Frequency	Smoking: Smoke Frequency	0 = Not at all; 2 = Some days; 2 = Every day; Missing = Skip; Missing = Prefer not the answer*	0-2
**Lifestyle (PXS)**	Smoking- Tobacco Smoking Status	Tobacco smoking status	0 = Never smoker; 1 = Former smoker; 2 = Current every day	0-2

			smoker; 2 = Current some day smoker; Missing = No matching concept; Missing = Light tobacco smoker; Missing = Heavy tobacco smoker*	
**SDOH (PXS)**	Income	Total combined household income range in last year	0 = <10K; 1 = 10-25K; 2 = 25-35K; 3 = 35-50K; 4 = 50-75K; 5 = 75-100K; 6 = 100-150K; 7 = 150-200K, 8 = >200K, Missing = Skip	0-8
**SDOH (PXS)**	Education	Education Level: Highest Grade	0 = Never attended; 1 = One through four; 2 = Five through eight; 3 = Nine through twelve; 4 = Twelve or GED; 5 = College one to three; 6 = College graduate; 7 = Advanced degree; Missing = Skip; Missing = Prefer not to answer	0-7
**SDOH (PXS)**	Neighborhood- Abandoned Buildings	How much you agree or disagree that there are lot of abandoned buildings in your neighborhood?	0 = Strongly agree; 1 = Agree; 2 = Disagree; 3 = Strongly disagree; Missing = Skip	0-3
**SDOH (PXS)**	Neighborhood- Alcohol Use	How much you agree or disagree that there is too much alcohol use in your neighborhood?	0 = Strongly agree; 1 = Agree; 2 = Disagree; 3 = Strongly disagree; Missing = Skip	0-3
**SDOH (PXS)**	Neighborhood- A lot of Crime	How much you agree or disagree that there is a lot of crime in your neighborhood?	0 = Strongly agree; 1 = Agree; 2 = Disagree; 3 = Strongly disagree; Missing = Skip	0-3

**SDOH (PXS)**	Neighborhood- Cleanliness	How much you agree or disagree that your neighborhood is clean?	0 = Strongly disagree; 1 = Disagree; 2 = Agree; 3 = Strongly agree; Missing = Skip	0-3
**SDOH (PXS)**	Neighborhood- Crime Rate Makes it Unsafe to Walk at Night	The crime rate in my neighborhood makes it unsafe to go on walks at night [PhenX]	0 = Strongly agree; 1 = Agree; 2 = Disagree; 3 = Strongly disagree; Missing = Skip	0-3
**SDOH (PXS)**	Neighborhood- Crime Rate Makes it Unsafe to Walk During the Day	The crime rate in my neighborhood makes it unsafe to go on walks during the day. Would you say that you…	0 = Strongly agree; 1 = Somewhat agree; 2 = Somewhat disagree; 3 = Strongly disagree	0-3
**SDOH (PXS)**	Neighborhood- Drug Use	How much you agree or disagree that there is too much drug use in your neighborhood?	0 = Strongly agree; 1 = Agree; 2 = Disagree; 3 = Strongly disagree; Missing = Skip	0-3
**SDOH (PXS)**	Neighborhood- Facilities to Bike	There are facilities to bicycle in or near my neighborhood, such as special lanes, separate paths or trails, shared use paths for cycles and pedestrians [PhenX]	0 = Strongly disagree; 1 = Somewhat disagree; 2 = Somewhat agree; 3 = Strongly agree; Missing = Skip	0-3
**SDOH (PXS)**	Neighborhood- Free/Low-Cost Recreation Facilities	My neighborhood has several free or low-cost recreation facilities, such as parks, walking trails, bike paths, recreation centers, playgrounds, public swimming pools, etc. Would you say that you…	0 = Strongly disagree; 1 = Somewhat disagree; 2 = Somewhat agree; 3 = Strongly agree; Missing = Skip; Missing = Don't Know	0-3
**SDOH (PXS)**	Neighborhood- Graffiti	How much you agree or disagree that there is a lot of graffiti in your neighborhood?	0 = Strongly agree; 1 = Agree; 2 = Disagree; 3 = Strongly disagree; Missing = Skip	0-3
**SDOH (PXS)**	Neighborhood- Get Along with Neighbors	People in this neighborhood generally don't get along with each other [PhenX]	0 = Strongly agree; 1 = Agree; 2 = Neither agree nor disagree; 3 =	0-4

			Disagree; 4 = Strongly disagree; Missing = Skip	
**SDOH (PXS)**	Neighborhood-Main Type of Housing	What is the main type of housing in your neighborhood [PhenX]	0 = Apartments or condos of more than 12 stories; 1 = Apartments or condos of 4-12 stories; 2 = Mix of single-family residences and townhouses, row houses, apartments, or condos; 3 = Duplex/townhouse; 4 = Detached single-family housing; Missing = Skip	0-4
**SDOH (PXS)**	Neighborhood-Neighbors Can Be Trusted	People in this neighborhood can be trusted [PhenX]	0 = Strongly disagree; 1 = Disagree; 2 = Neither agree nor disagree; 3 = Agree; 4 = Strongly agree; Skip = Missing	0-4
**SDOH (PXS)**	Neighborhood-Neighbors Take Good Care of Their Homes	How much you agree or disagree that people in your neighborhood take good care of their houses and apartments?	0 = Strongly disagree; 1 = Disagree; 2 = Agree; 3 = Strongly agree; Missing = Skip	0-3
**SDOH (PXS)**	Neighborhood-Neighbors Watch Out for Each Other	How much you agree or disagree that in your neighborhood people watch out for each other?	0 = Strongly disagree; 1 = Disagree; 2 = Agree; 3 = Strongly agree; Missing = Skip	0-3
**SDOH (PXS)**	Neighborhood-Noise	How much you agree or disagree that your neighborhood is noisy?	0 = Strongly agree; 1 = Agree; 2 = Disagree; 3 = Strongly disagree; Missing = Skip	0-3
**SDOH (PXS)**	Neighborhood-People Share the Same Values	How much you agree or disagree that people in your neighborhood share the same values?	0 = Strongly disagree; 1 = Disagree; 2 =	0-4

			Neither agree nor disagree; 3 = Agree; 4 = Strongly agree; Skip = Missing	
**SDOH (PXS)**	Neighborhood- Safe from Crime	My neighborhood is safe from crime [PhenX]	0 = Strongly disagree; 1 = Disagree; 2 = Agree; 3 = Strongly agree; Missing = Skip	0-3
**SDOH (PXS)**	Neighborhood- Shops, Stores, Markets or Other Places to Buy Things are Within Walking Distance	Many shops, stores, markets, or other places to buy things I need are within easy walking distance of my home [PhenX]	0 = Strongly disagree; 1 = Somewhat disagree; 2 = Somewhat agree; 3 = Strongly agree; Missing = Skip	0-3
**SDOH (PXS)**	Neighborhood- Sidewalks on Most Streets	There are sidewalks on most of the streets in my neighborhood [PhenX]	0 = Strongly disagree; 1 = Somewhat disagree; 2 = Somewhat agree; 3 = Strongly agree; Missing = Skip	0-3
**SDOH (PXS)**	Neighborhood- Too Many People Hanging Around on the Streets Near Home	How much you agree or disagree that there are too many people hanging around on the streets near your home?	0 = Strongly agree; 1 = Agree; 2 = Disagree; 3 = Strongly disagree; Missing = Skip	0-3
**SDOH (PXS)**	Neighborhood- Transit Stop Within Walking Distance	It is within a 10–15-minute walk to a transit stop, such as bus, train, trolley, or tram, from my home [PhenX]	0 = Strongly disagree; 1 = Somewhat disagree; 2 = Somewhat agree; 3 = Strongly agree; Missing = Skip	0-3
**SDOH (PXS)**	Neighborhood- Trouble with Neighbors	How much you agree or disagree that you are always having trouble with your neighbors?	0 = Strongly agree; 1 = Agree; 2 = Disagree; 3 = Strongly disagree; Missing = Skip	0-3
**SDOH (PXS)**	Neighborhood- Vandalism	How much you agree or disagree that vandalism is common in your neighborhood?	0 = Strongly agree; 1 = Agree; 2 = Disagree; 3 = Strongly disagree; Missing = Skip	0-3

Supplementary Table 2: All of Us Percentage of Non-Missing Data for Each Variable.

Category (Risk Score)	Risk Factor	Percent Non-Missing
**Outcome**	HF	100.00%
**Covariate**	Age	100.00%
**Covariate**	Sex	100.00%
**PGS**	PGS	100.00%
**Clinical (CRS)**	Diastolic Blood Pressure	92.97%
**Clinical (CRS)**	Glucose	53.29%
**Clinical (CRS)**	HbA1c	32.50%
**Clinical (CRS)**	HDL cholesterol	36.84%
**Clinical (CRS)**	LDL cholesterol	37.57%
**Clinical (CRS)**	Systolic Blood Pressure	92.98%
**Clinical (CRS)**	T2D	97.85%
**Clinical (CRS)**	Triglycerides	37.05%
**Lifestyle (PXS)**	BMI	95.86%
**Lifestyle (PXS)**	Physical Activity- Everyday Physical Activity	94.49%
**Lifestyle (PXS)**	Smoking	98.42%
**SDOH (PXS)**	Annual Income	79.98%
**SDOH (PXS)**	Census Median Income	96.10%
**SDOH (PXS)**	Highest Education	97.78%
**SDOH (PXS)**	Neighborhood- Abandoned Buildings	42.71%
**SDOH (PXS)**	Neighborhood- Alcohol Use	41.29%
**SDOH (PXS)**	Neighborhood- A lot of Crime	43.19%
**SDOH (PXS)**	Neighborhood- Cleanliness	42.15%
**SDOH (PXS)**	Neighborhood-Crime Rate Makes It Unsafe to Walk at Night	41.54%
**SDOH (PXS)**	Neighborhood- Crime Rate Makes It Unsafe to Walk During the Day	39.33%
**SDOH (PXS)**	Neighborhood- Drug Use	42.55%
**SDOH (PXS)**	Neighborhood- Facilities to Bike	40.95%
**SDOH (PXS)**	Neighborhood- Free/Low-Cost Recreation Facilities	37.27%
**SDOH (PXS)**	Neighborhood- Get Along with Neighbors	43.48%
**SDOH (PXS)**	Neighborhood- Graffiti	43.66%
**SDOH (PXS)**	Neighborhood-Main Type of Housing	43.34%
**SDOH (PXS)**	Neighborhood- Neighbors Can Be Trusted	43.03%
**SDOH (PXS)**	Neighborhood- Neighbors Take Good Care of Their Homes	43.20%

**SDOH (PXS)**	Neighborhood- Neighbors Watch Out for Each Other	43.15%
**SDOH (PXS)**	Neighborhood- Noise	43.40%
**SDOH (PXS)**	Neighborhood- People Share the Same Values	42.65%
**SDOH (PXS)**	Neighborhood- Safe from Crime	43.10%
**SDOH (PXS)**	Neighborhood- Shops, Stores, Markets or Other Places to Buy Things are Within Walking Distance	43.49%
**SDOH (PXS)**	Neighborhood- Sidewalks on Most Streets	41.91%
**SDOH (PXS)**	Neighborhood- Too Many People Hanging Around on the Streets Near Home	43.08%
**SDOH (PXS)**	Neighborhood- Transit Stop Within Walking Distance	41.45%
**SDOH (PXS)**	Neighborhood- Trouble with Neighbors	43.30%
**SDOH (PXS)**	Neighborhood- Vandalism	43.06%
**SDOH (PXS)**	Social Deprivation Index	96.10%
